# A Timely Review of the Genetics of Epileptic Encephalopathies

**DOI:** 10.15844/pedneurbriefs-29-11-3

**Published:** 2015-11

**Authors:** Jeremy L. Freeman

**Affiliations:** 1 Department of Neurology, The Royal Children's Hospital, Melbourne VIC Australia

**Keywords:** Epileptic Encephalopathy, Childhood, Genetics, Phenotype

## Abstract

Investigators from UCL Institute of Child Health, London and The University of Melbourne reviewed current knowledge of the genetics of epileptic encephalopathies of infancy and childhood.

Investigators from UCL Institute of Child Health, London and The University of Melbourne reviewed current knowledge of the genetics of epileptic encephalopathies of infancy and childhood. [1]

COMMENTARY. In an era of expanding availability of clinical genetic testing and a choice of technologies with which to perform this testing, child neurologists need to have a good understanding of the diagnostic methods used and the capacity to make good clinical sense of the results.

This review of the genetic ‘lansdcape’ of epileptic encephalopathy starts with discussion of the concept of epileptic encephalopathy and an acknowledgement that the relative contribution of developmental and epileptic mechanisms to encephalopathy are difficult to tease out. The authors then discuss the different technologies including chromosomal microarray, next generation parallel sequencing of multiple genes and whole exome sequencing, providing information about their relative strengths and weaknesses and the current yield of these investigations.

The authors describe considerations of heredity and genetic mosaicism that are critical to genetic counselling of families and explain common clinical and research scenarios.

Phenotypic heterogeneity or pleiotropy, whereby mutation in one gene can result in a number of different phenotypes (including no disease) is discussed, as too is genetic heterogeneity, whereby one electroclinical syndrome can result from mutation in any of a number of genes. Insights into the neurobiology of severe epilepsies and translation of these insights into patient care are discussed in terms of future diagnostic approaches and future treatment approaches.

The figures and tables provide more detail about each of the electroclinical syndromes that comprise the epileptic encephalopathies, the proportion of children with specific gene mutations, the ages of onset of the syndromes and the neurobiology summarized in a neuron-synapse cartoon. The supplementary appendix contains some pearls in relation to clinical features, imaging findings and treatments for patients with certain gene mutations. Overall, a nicely written and illustrated summary of a difficult topic.

Another resource that may be of interest to child neurologists and epileptologists is ‘The Epilepsiome’. Part of the ILAE Genetic Commission blog, this website is an epilepsy genetics “wiki” that has useful summaries of important epilepsy genes [2].
